# Introduction of Nicotine Analogue-Containing Oral Pouch Products in the United States

**DOI:** 10.21203/rs.3.rs-5110349/v1

**Published:** 2024-09-20

**Authors:** Sven E. Jordt, Sairam V. Jabba

**Affiliations:** Duke University School of Medicine; Duke University School of Medicine

**Keywords:** Addiction, Non-cigarette tobacco products, Tobacco Industry, Nicotine, Nicotine analogue

## Abstract

**Background:**

In 2023, 6-methyl nicotine (6MN), a synthetic nicotine analogue, was introduced in US-marketed electronic cigarette products advertised as exempt from regulation. It is unknown whether the use of 6MN has spread to the oral nicotine pouch product category that has become increasingly popular.

**Methods:**

Industry reports and the USPTO database were searched for informationon 6-methyl nicotine products. The search terms “Metatine”, “Nixotine”, “Imotine” and “pouches” were shortlisted and used to identify US-marketed pouch products. Ingredient contents were compared to popular products, and safety warnings and regulatory statements assessed in context with US state and federal regulations.

**Results:**

Two US-based brands, “MG” and “Hippotine” pouches, were identified in August 2024, advertised to contain “Imotine”-trademarked 6MN. MG Pouches are marketed in four youth-appealing flavors. “Hippotine”-branded pouches are marketed in two flavors. 6MN contents ranged between 8mg - 25mg with almost identical ingredient lists otherwise. Products list extensive addiction and health warnings, including warnings not to operate vehicles. Vendors state that these are not a tobacco product, implicating that federal and state tobacco regulations do not apply.

**Conclusions:**

The spread of nicotine analogues to additional product categories such as oral pouches is concerning, especially given the high 6MN contents that exceed nicotine contents in popular US-marketed oral nicotine pouch products. Legislators and regulators need to provide certainty about the regulatory status of nicotine analogues to prevent further erosion of tobacco flavor bans and other regulations.

## Introduction

In 2023, 6-methyl nicotine (6MN), a synthetic nicotine analogue, was introduced in US-marketed electronic cigarette products advertised as exempt from FDA regulation [1]. Since then, several disposable e-cigarette products and refill liquids containing 6MN appeared on the US market, with the compound branded “Metatine” or “Nixotine”, the latter mixed with nicotinamide [2]. An additional trademark, “Imotine”, was registered for 6MN, marketed by the company Novel Compounds [3–5].

Oral nicotine pouches (ONP) represent a new tobacco product category with rapidly growing sales in the United States and worldwide [6]. It is unknown whether the use of 6MN has spread to this product category.

## Methods

Industry reports, Google Patents and the USPTO database were searched for information, patent applications and trademarks for 6-methyl nicotine products. The search terms “Metatine”, “Nixotine”, “Imotine”, “6-methyl nicotine” (and variations thereof) and “pouches” were shortlisted and used to identify US-marketed pouch products. Products were purchased from web merchants to confirm availability. Ingredient contents were reviewed and compared with ingredients in market-leading Oral Nicotine Pouch products. Marketing and safety claims were reviewed and regulatory statements assessed in context with regulations of US states, especially California, and federal laws and regulations.

## Results and Discussion

We used the search terms “Metatine”, “Nixotine” or “Imotine” and “pouch” to search for 6MN-containing oral pouch products marketed by web merchants. In August 2024, we identified two US-based brands, “MG” and “Hippotine” pouches, advertised to contain “Imotine”. No “Metatine”, or “Nixotine” pouch products were identified. “MG” pouches are marketed by Upperdeckys.com, a vendor of caffeine-containing “Energy” pouches. “Hippotine” pouches are marketed by the web merchant Happyhippo.com, a vendor of Kratom, an herbal extract containing opioid receptor agonists [7 8].

MG Pouches are marketed in four flavors, Cool Mint (8 mg Imotine per pouch), Buzzin Berry (8 mg), Wintergreen (15 mg) and Orange Creamsicle (25 mg) (Table 1). Hippotine-branded pouches are marketed in two flavors, Guava Juice (15 mg Imotine per pouch) and Wintergreen (25 mg) (Table 1). The listed 6-methyl nicotine contents of the newly introduced products are either identical (8 mg), or by far exceed (15mg, 25mg), the maximal nicotine contents of the most popular US-marketed nicotine pouch products (Zyn, Velo, On!). Both brands provide almost identical ingredient lists on the back of the cans, containing “Coconut Fiber” (MG) or “Coconut Coir” (Hippotine), “Vegetable Glycerin, Palm Oil, Xylitol, Natural Flavor, Water, Imotine^™^, Sodium Carbonate, Xanthan Gum, Stevia, Salt”, suggesting they are produced by the same manufacturer.

Extensive addiction and health warning are provided:

“Imotine^™^ is chemically distinct from nicotine. It may still be addictive, may have toxicity profile similar to Nicotine, and should only be used by current adult tobacco users and never by minors (Persons under the age of 21).” [8]

**Figure 1:**
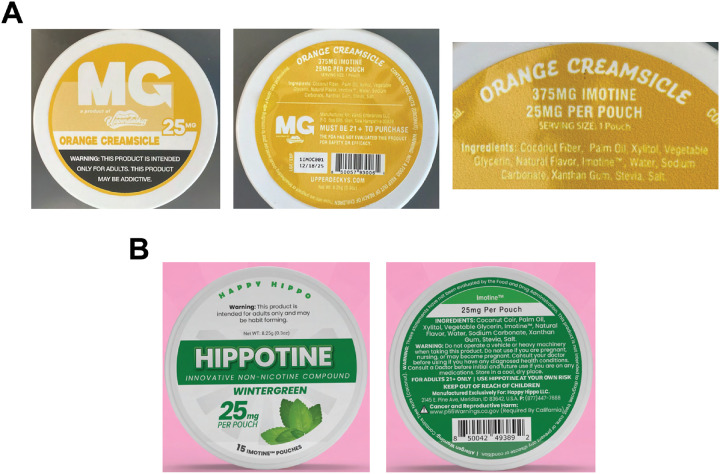
Oral pouch products containing “Imotine”-branded 6-methyl nicotine **A**. Photographs of Orange Creamsicle-flavored “MG” pouches can purchased by the authors, labelled to contain 25mg “Imotine” per pouch. Left: Front of can, Middle: Back of can, Right: “Imotine” content, ingredients list. **B**. Wintergreen-flavored “Hippotine” pouches can, labelled to contain 25mg “Imotine”. Left: Front of can, Right: Back of can.

“Do not use if you are pregnant, nursing or may become pregnant. Consult your doctor before using if you have any diagnosed health conditions. Consult a Doctor before initial and future use if you are on any medications.” (Fig. 1)

“Use Hippotine at your own risk.” (Fig. 1)

Additionally, a warning usually not associated with tobacco products is included: “Warning: Do not operate a vehicle or heavy machinery when taking this product. “ (Fig. 1)

Both brands state, either on their website (MG) or on the can (Hippotine) that “This product is not intended to diagnose, treat, cure, or prevent any disease or condition.”, likely to pre-empt regulation of the products by FDA as drugs (Fig. 1) [8]. The vendor also states that “Imotine^™^ is not considered a “tobacco product”.”, suggesting that tobacco regulatory restrictions do not apply [9]. Hippotine pouches are advertised as “Available to Californians (not subject to flavored pouch restriction)”, aiming to undermine California’s ban on characterizing flavors that extends to oral nicotine pouches [7].

## Conclusions

The spread of nicotine analogues to additional product categories such as oral pouches is concerning, especially because of the high listed 6MN contents of the newly introduced products. Given the higher potency of 6MN compared to nicotine in pharmacological studies, regulators need to rapidly assess the potential public health threats associated with these products [1]. Legislators and regulators also need to provide certainty about the regulatory status of nicotine analogues to prevent further erosion of flavor bans and other regulations.

## Figures and Tables

**Table 1:** Brands, 6MN trademarks, flavors and listed 6MN contents in US-marketed oral pouch products

Brand	6MN Trademark	Flavor	6MN Strength/pouch
MG	Imotine	Cool Mint	8 mg
		Buzzin Berry	8 mg
		Wintergreen	15 mg
		Orange Creamsicle	25 mg
Hippotine	Imotine	Guava Juice	15 mg
		Wintergreen	25 mg
